# Gender-Based Performance in Mathematical Facts and Calculations in Two Elementary School Samples From Chile and Spain: An Exploratory Study

**DOI:** 10.3389/fpsyg.2021.703580

**Published:** 2021-08-17

**Authors:** Violeta Pina, Diana Martella, Salvador Chacón-Moscoso, Mahia Saracostti, Javier Fenollar-Cortés

**Affiliations:** ^1^Departamento de Psicología Evolutiva y de la Educación, Facultad de Educación, Economía y Tecnología de Ceuta, Universidad de Granada, Ceuta, Spain; ^2^Instituto de Estudios Sociales y Humanísticos, Facultad de Ciencias Sociales y Humanidades, Universidad Autónoma de Chile, Región Metropolitana, Chile; ^3^Departamento de Psicología Experimental, Universidad de Sevilla, Sevilla, Spain; ^4^Departamento de Psicología, Universidad Autónoma de Chile, Santiago, Chile; ^5^Núcleo Científico y Tecnológico en Ciencias Sociales y Humanidades, Universidad de la Frontera, Temuco, Chile; ^6^Escuela de Trabajo Social, Facultad de Ciencias Sociales, Universidad de Valparaíso, Valparaíso, Chile; ^7^Departamento de Psicología, Universidad Loyola Andalucía, Sevilla, Spain

**Keywords:** gender differences, primary education, mathematical fluency, calculation, children

## Abstract

Gender differences in mathematical performance are not conclusive according to the scientific literature, although such differences are supported by international studies such as the Trends in International Mathematics and Science Study (TIMSS). According to TIMSS 2019, fourth-grade male students outperformed female students in Spanish-speaking countries, among others. This work approaches the study on gender difference by examining the basic calculation skills needed to handle more complex problems. Two international samples of second and third graders from Chile and Spain were selected for this exploratory study. Tests on basic mathematical knowledge (symbolic and non-symbolic magnitude comparisons, fluency, and calculation) were administered. The tests did not show significant difference or size effect between genders for mean performance, variance in the distribution of performance, or percentiles. As noted in the existing literature on this topic and reiterated by these findings, great care should be exercised when reporting on possible gender differences in mathematical performance, as these can contribute to low self-concept among female students.

## Introduction

As technology grows by leaps and bounds, new fields of study and analysis tools are also expanding: Big Data, artificial intelligence, modeling engineering, software architecture, etc. This has increased the demand for professionals, both men and women, with the solid knowledge of mathematics needed for such roles (Belloum et al., 2019). However, women continue to be underrepresented in this kind of professional roles but overrepresented in lower paying jobs (Adams et al., 2019). Determining the origin of these differences is challenging as cognitive, social, and cultural factors over the course of one's life may all contribute, making it difficult to connect mathematical abilities during childhood to gender differences on the job market. However, if this gender gap were intrinsic, it could appear during childhood, making it essential to determine whether this is the case and, thus, intervene to avoid future underrepresentation of women.

The reduced presence of women in mathematical fields is nothing new. As indicated by Kane and Mertz (2012, p. 10), differences between males and females have been found in the mathematics participation rate, mean and high-end performance, and variance in the distribution of performance, making gender difference the subject of extensive debate. Havelock Ellis (1894) was the first to intend to empirically justify the differences between men and women with his variability hypothesis. According to this hypothesis, men could be expected to show greater variability than women in physical and intellectual abilities. This would explain the predominance of men in higher academic and scientific positions, but also their prevalence in crime. This hypothesis coincided with the expanded presence of women in higher education and their demands for recognition (Shields, 1982) and continued to be defended, with minor modifications, until the 21st century, when the focus turned to extreme scores and percentiles. In particular, some studies found more females in the lower percentiles and more males in the higher percentiles for different math assignments (Barbaresi et al., 2005; Reigosa-Crespo et al., 2012; Anaya et al., 2017).

Nevertheless, in a wide study carried out by Baker and Jones (1993) with 77,602 students from 19 countries, mathematical performance between genders was found to vary by country and the type of education students received; additionally, the differences were noted to be decreasing over time. The authors also largely debunked the hypothesis of variability when they pointed out that gender differences in performance decrease when women have more equitable access to higher education and qualified work. Indeed, several studies (for instance, Kane and Mertz, 2012) have moved away from the deterministic hypothesis of variability, providing empirical evidence for a hypothesis of sociocultural factors.

Coinciding with the argument that sociocultural factors have the greatest bearing on mathematical performance, differences between genders should gradually disappear as society becomes more progressive and inclusive. Lindberg et al. (2010) support this hypothesis in a broad meta-analysis. Based on 242 studies published between 1999 and 2007 (a total sample of 1,286,350 individuals both males and females), the authors concluded that mathematical performance is similar for both genders, noting that any gender difference can be attributed to unrelated factors. The authors conclude that it is crucial to disseminate these results to counteract stereotypes about [alleged] female math inferiority (Lindberg et al., 2010, p. 1134). More recent studies have replicated these results (for instance, Voyer and Voyer, 2014; Scheiber et al., 2015; Kersey and Cantlon, 2018), and others have limited the differences to a minority of tasks (Hutchison et al., 2019).

Given the empirical evidence reported in the literature, males and females would be expected to perform similarly on international studies such as the TIMSS (Instituto Nacional de Evaluación Educativa [INEE], 2016, 2020). Unfortunately, this is not the case. According to the TIMSS, the gender gap in mathematical performance is present as early as fourth grade and, according to the last edition (2019), has even increased in countries such as Spain. Gender differences at the fourth grade level can also be noted, although to a lesser extent, in other Spanish-speaking countries that participated in TIMSS 2019. Chile, for example, went from having no significant gender differences in mathematics in the 2015 report to having significant ones in the 2019 report (Instituto Nacional de Evaluación Educativa [INEE], 2016, 2020). In fact, samples from Spanish-speaking countries show a large gender gap in international reports, but few studies include them in their analysis (e.g., the meta-analysis by Voyer and Voyer, 2014).

This exploratory study considers two Spanish-speaking samples from Chile and Spain, both of which are underrepresented in the literature. First, it is important to analyze potential gender differences for these countries. Second, by looking into the samples from these two countries, common patterns may be found that could allow an early intervention to be designed.

Both Spain and Chile have similar math curricula at the elementary school level. In both Chile (MINEDUC, 2018) and Spain (e.g., Decreto n.°, 198/2014), students are doing single-digit addition and subtraction at the end of the first grade (at around age seven). In second grade, they learn multiplication and begin adding and subtracting beyond single digits. In third grade, they begin using multiplication and division to solve math problems. Although both samples are socially and culturally distinct, the objective of this study was to investigate whether gender differences exist and, if so, whether the pattern is the same for the two Spanish-speaking countries. Since both Chile and Spain have similar math curricula, if this was the case, the gender gap could be considered intrinsic.

International evaluations and even math tests administered at schools often do not consider the skills and basic knowledge for acquiring mathematical abilities, and are instead focused on complex calculation or problem solving (Nosworthy et al., 2013). Hence, when gender differences are observed in such tests, it is hard to determine whether such differences are also present for more basic math skills. A logical hypothesis is that the basic abilities needed for complex tasks would also show a gender gap. In recent years, different studies have explored what basic numerical skills necessary for mathematical achievement are responsible for gender differences (for instance, Kersey and Cantlon, 2018; Hutchison et al., 2019).

In this regard, the literature has widely reported on the basic skills required for mathematical achievement. On one hand, non-symbolic magnitude comparison (e.g., the ability to look at two groups of objects and determine which is greater in number), is considered a stepping stone to learning numbers (see Landerl, 2019). For non-symbolic comparison, visuospatial perception, not the ability to count, is required (Kersey and Cantlon, 2018). Once it is acquired, boys and girls can relate quantities with symbolic numbers (for instance, relating the number four with four objects) and learn the meaning of the Arabic numeral. On the other hand, symbolic magnitude comparison (the ability to choose which of two numbers is larger) requires participants to efficiently access the analog magnitude representations that correspond to the Arabic numerals in order to determine which number is greater (Landerl, 2019). Two tasks are widely used to evaluate these abilities: the symbolic magnitude comparison task and the non-symbolic magnitude comparison task. A recent meta-analysis has confirmed that both tasks correlate with subsequent mathematical performance, although the correlation is higher for the symbolic comparison task (Schneider et al., 2016).

Once children understand the meaning of the Arabic numerals, they start to count. The first operations are simple single-digit addition and subtraction with a sum of <10. Depending on one's knowledge of arithmetic facts, two strategies are mainly used. In the beginning, the child will have to count to solve the operation; and through repetition, the answers become lodged in their memory (De Smedt et al., 2019). The automation of these operations has been considered critical to developing robust complex calculations (Royer et al., 1999).

Calculation and mathematical problems are considered more complex areas of mathematical performance. Mathematical problems also require other abilities such as reading comprehension (Abedi and Lord, 2001; Donlan et al., 2007). Calculation usually includes the four basic mathematical operations (addition, subtraction, multiplication, and division) and generally cannot be solved from memory, since it usually involves double digits or more. Children can use a wide variety of strategies for approaching these operations, such as decomposition or sequencing (Hickendorff et al., 2019). Calculation also requires executive functions, such as working memory. The strategy children use depends not only on their individual skills but also on the instruction they have received. Gender differences have been found (e.g., Diamantopoulou et al., 2012) for these complex tasks. Winkelmann et al. (2008) proposed that disparities in different studies on gender differences can be attributed to what is considered “basic ability” in a particular study and on the cognitive resources required for each task. The breakdown of tasks by gender differences allows for the design of learning and support strategies in specific areas to gradually reduce the gender gap.

Gender differences tend to be more evident in upper elementary. Some studies do present evidence of a gender gap for lower elementary (Jordan et al., 2006; Scheiber et al., 2015), but others do not (Lachance and Mazzocco, 2006). Third grade seems to be a critical year for the detection of gender differences in mathematical performance, although the studies at this level are scarce. As an example, Germany presents gender differences starting in third grade (Winkelmann et al., 2008). In Spain, the TIMSS (2015 and 2019) reports a large gender gap in fourth grade, thus suggesting a possible gender gap in third grade as well. Another international study, TERCE 2015 (UNESCO, 2016), shows that the number of Latin American countries where males have an advantage over females increases between third and sixth grade. Therefore, second and third grade are considered crucial. The aim of this study is not to determine the age at which gender differences, if they exist, appear, but to observe whether these differences are detected in lower elementary in Spanish-speaking countries.

This study delves into these differences to determine if they are present in the first years of school for basic math skills. After controlling for reading proficiency, mathematical performance by Spanish-speaking second and third graders in Chile and Spain will be compared for non-symbolic comparison, symbolic comparison, fluency, and calculation. As part of this study, any differences in mean performance, variance in the distribution of performance, and percentiles will be assessed. According to the literature, differences are not expected in basic mathematical tasks such as symbolic and non-symbolic comparison but could appear for tasks that require mathematical fluency and calculation. We hypothesize that a greater number of males will be in the higher percentiles and a greater number of females will be in the lower percentiles.

## Methodology

### Participants

The participants were second and third graders at four schools in the O'Higgins region (Chile) and seven schools in Murcia (Spain). These children were organized into two separate samples, one for each country, with a mean age of 7.75 for the second graders (*M* = 7.68, *SD* = 0.31 for the Spanish children, and *M* = 7.88, *SD* = 0.48 for the Chilean children) and 8.77 for the third graders (*M* = 8.7, *SD* = 0.33 for the Spanish children, and *M* = 8.89, *SD* = 0.39 for the Chilean children). The sample was formed by 201 Spanish school children (107 second graders and 94 third graders; 55.2% male and 44.8% female) and 184 Chilean school children (90 second graders and 94 third graders; 48.9% male and 51.1% female) and was collected through incidental sampling. The following exclusion criteria were established: vision difficulties, diagnosis of autism spectrum disorder or cognitive impairment, insufficient knowledge of the Spanish language, and, in the case of the third graders, significant difficulties reading. Table 1 details the number of males and females by grade and country. Possible differences regarding age (Mann–Whitney U) and gender proportion by grade and country (χ^2^) were examined.

**Table 1:** Distribution in the Spanish language sample of second and third graders: scoring on symbolic test by gender.

	**Spanish sample**					**Chilean sample**				
***M*(*DT*)**	**Male**	**Female**	***U***	***p***	***r^a^***	**Male**	**Female**	***U***	***p***	***r***
Second grade										
N	107					90				
Gender *n* (%)	56(52.3)	51(47.7)				49(54.4)	41(45.6)			
Magnitude comparison										
Symbolic	29.3(9.3)	33.0(6.8)	1055.0	0.020	−0.26	29.9(10.1)	30.9(10.6)	961.0	0.727	
Num. errors	0.1(0.4)	0.1(0.9)	1572.2	0.060		0.6(1.3)	0.7(1.2)	931.5	0.850	
Non-symbolic	29.1(7.6)	31.6(5.7)	1103.5	0.042	−0.23	28.8(13.8)	33.9(12.0)	796.5	0.092	
Num. errors	0.3(1.0)	0.6(0.1)	1419.5	0.932		1.4(2.7)	2.2(2.9)	826.0	0.110	
Third grade										
N	94					94				
Gender *n* (%)	55(58.5)	39(41.5)				55(58.5)	39(41.5)			
Magnitude comparison										
Symbolic	37.7(7.0)	39.8(6.2)	837.0	0.069		39.3(10.2)	37.4(11.2)	1158.0	0.513	
Num. errors	0.2(0.6)	0.1(0.4)	1126.5	0.443		0.6(1.1)	0.6(1.1)	1051.5	0.847	
Non-symbolic	32.3(7.3)	31.7(4.8)	1196.0	0.344		37.0(11.3)	35.5(11.4)	1193.5	0.355	
Num. errors	0.5(1.0)	0.3(0.6)	1135.5	0.412		1.7(2.7)	1.1(1.6)	1133.5	0.618	
PreDisCal										
Sentences	13.6(14.7)	14.7(2.9)	905.5	0.199		13.2(6.9)	12.8(6.2)	1090.0	0.896	
Num. errors	2.2(1.9)	1.6(1.2)	1237.0	0.193		6.5(9.3)	3.2(2.4)	1252.5	0.163	
Mathematical fluency	17.7(6.8)	17.1(4.5)	1079.5	0.960		12.9(5.5)	10.7(5.1)	1369.5	0.023	0.27
Num. errors	1.5(2.5)	1.1(1.7)	1127.0	0.652		1.8(2.6)	1.3(1.4)	1050.5	0.864	
Calculation	11.6(4.2)	11.0(3.4)	1143.0	0.589		9.0(4.3)	9.0(4.3)	1126.0	0.683	
Num. errors	0.2(0.6)	0.3(0.6)	1202.0	0.313		4.1(8.0)	1.9(8.0)	1294.5	0.083	

*Scores are shown in terms of mean and standard deviation (not mean scores) to facilitate understanding*.

a *Effect size corresponding to the biserial correlation between ranges. Only values corresponding to significant differences between the groups are provided*.

This study was carried out following the recommendations of the Chilean Commission for Scientific and Technological Investigation (CONICYT in its Spanish acronym). The protocol was approved by the Ethics Committee of Universidad de la Frontera (Act 066-2017, on Sheet 036-17). The study complied with the standards of the ethical committee of Universidad Autónoma de Chile and with the agreement between the Department of Education and Universities of the autonomous community of Region of Murcia and Universidad de Murcia. Informed consent was requested to participate in this study.

### Instruments

The following paper-and-pencil tests were administered to measure mathematical performance and reading fluency.

Numerical magnitude comparisons (Nosworthy et al., 2013). This task includes two different parts:

- Symbolic task (56-digit pairs), where the participants were asked to compare numerical pairs (numbers one through nine) and indicate which number was higher.
- Non-symbolic task (56 dot arrays), where dot arrays of varying quantities are present and the participants were asked to indicate which side had more dots.

Each test had a time limit of 1 min, and the dependent variable was the number of correct answers.

PreDisCal (Pina et al., 2020). This is a set of three tests that measure reading fluency, mathematical fluency, and calculation, in that order. Although the PreDisCal scale was developed in Spain, a cross-validation process was carried out in several Latin American countries. Some of the items were modified because of interpretation issues (related to sociocultural factors) in Latin American countries. The test included the following tasks:

- Sentences. This task consists of 47 sentences that assess reading fluency. Each sentence has a missing word, for instance: “The strawberries are …” followed by five answer options (one is correct). The incorrect alternatives are close grammatically or semantically. All the sentences are easy to understand. The test had a time limit of 3 min. The dependent variable is the number of correct answers. It is a validated test with adequate test-retest reliability [*r*(169) = 0.8, *p* < 0.001].
- Mathematical fluency. The arithmetic facts are tested through 63 single-digit addition and subtraction operations. All sums are < 10. The maximum time is 1 min in this test, and the dependent variable is the number of correct answers. The reliability of the test is adequate [*r*(169) = 0.85, *p* < 0.001].
- Calculation. This test comprises 45 items of increasing difficulty and assesses complex calculation. Participants have to determine what number or symbol, or if both are missing in a comparison of two operations (e.g., “3 + _ = 5 + 1”). The test had a time limit of 3 min, and the dependent variable was the number of correct answers. The test-retest reliability of the validated scale was acceptable [*r*(169) = 0.75, *p* < 0.001].

### Procedure

The tasks were administered in February for Spain and in September of the same year for Chile in order to reflect the same moment of the academic calendar in both countries. Trained evaluators collectively administered the test during the school day. Given that PreDisCal required a certain degree of reading proficiency and complex calculation ability, it was administered only to the third graders, while the magnitudes comparison tests were administered to both the second and third grade samples. In the case of the magnitude comparisons, the symbolic test was administered first, followed by the non-symbolic test. The PreDisCal was administered in keeping with the order established for that test, e.g., sentences, mathematical fluency, and calculation.

### Data Analysis

First, the comparability of the groups was verified by assessing age and gender differences by grade and country (χ^2^), and mean performance in the sentences test between countries (Student t). Second, basic parametric assumptions for data analysis were verified (normality using the Kolmogorov–Smirnov test, homoscedasticity using the Levene test). Non-parametric tests were carried out using the Mann–Whitney U and statistical biserial correlations (*r* = 0.1 as low; *r* = 0.3 as medium, and *r* = 0.5 as high effect; Cohen, 1988); and χ^2^ to compare gender and percentile performance distribution in third grade.

## Results

Both samples showed a similar gender distribution of participants (χ^2^ = 0.09, *p* = 0.768 for second grade; χ^2^ = 0, *p* = 1 for second grade). There was no significant evidence between countries in relation to the mean performance in the sentences test (*t* = 1.35; *p* = 0.178). However, significant differences were detected between countries regarding the mean age of students (*t* = −5.04, *p* < 0.001, *d* = −0.52 for the third grade; *t* = −4.18, *p* < 0.001, *d* = −0.86 for the second grade), with moderate to high effect sizes (Cohen, 1988). Nevertheless, these differences in age are not relevant to this study, since the analysis between countries is carried out comparing gender, a variable for which there are no significant differences in the samples of both countries.

The distribution of data was examined for each dependent variable according to country and course. Except for the mathematical fluency and calculation tests in the Chilean third grade sample, the remaining variables followed a non-normal distribution (K-S < 0.05); thus, non-parametric analyses were applied.

**Figure 1:**
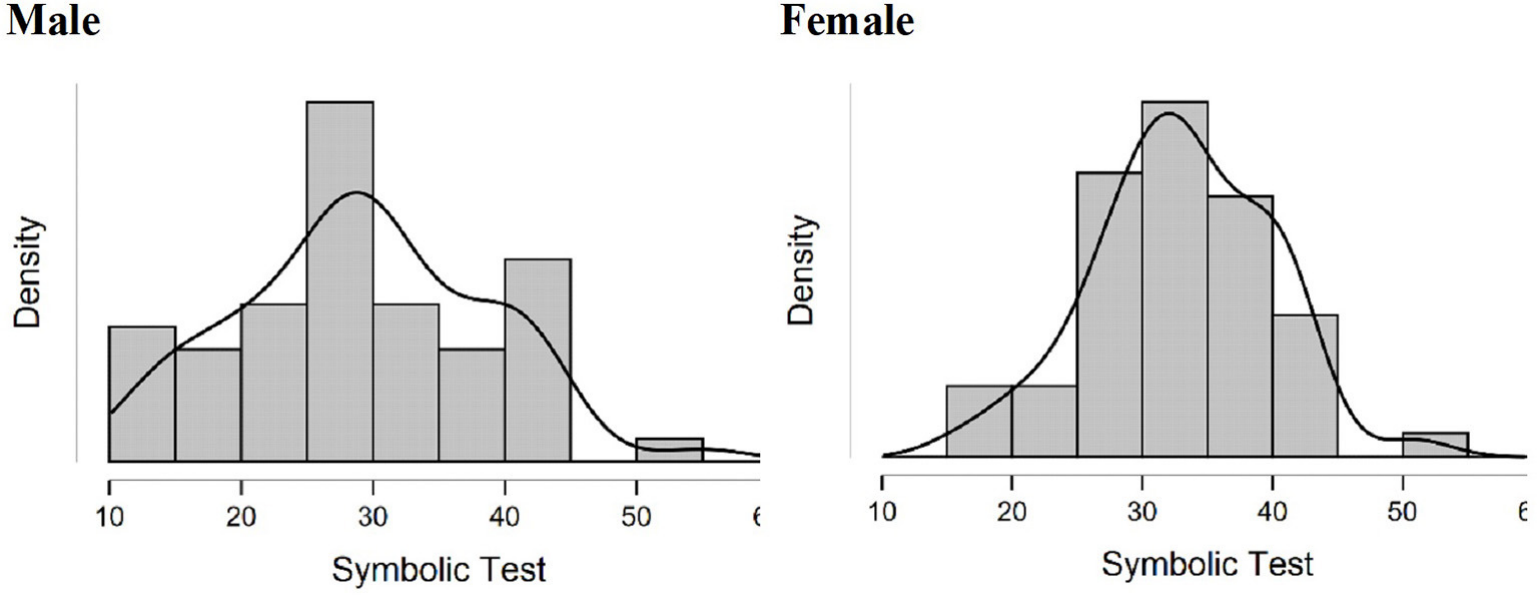
Distribution in the Spanish language sample of third grader scores on the symbolic test by gender.

The Levene test showed significant difference in variance between genders in the symbolic test for the Spanish sample of second graders (*F* = 3.97, *p* = 0.049). The scores of boys showed higher variability (σ^2^ = 86.5) with skewness of 0.18, (*SE* = 0.32) and kurtosis of −0.19 (*SE* = 0.63), compared with those of the girls (σ^2^ = 46.3) with skewness of 0.1 (*SE* = 0.33), and kurtosis of 0.27 (*SE* = 0.66), as shown in Figure 1. Differences in variance were not found for the other tests for either sample.

Spearman's correlation was carried out between tests for each country (Table 2). The correlation between scores on the symbolic and non-symbolic tests was crucially positive and high in the second grade sample. As for the third graders, mathematical fluency positively correlated to calculation and with sentences; these last two subscales correlated positively. The symbolic test also correlated positively with the non-symbolic one (*r*_s_ = 0.46, p < 0.001).

**Table 2:** Spearman's correlations between the PreDisCal and magnitude comparison (third grade tests).

	**PreDisCal**			**Magnitude comparison**
	**Sentences**	**Mathematical fluency**	**Calculation**	**Symbolic**
PreDisCal				
Mathematical fluency	0.194			
Calculation	0.253^*^	0.451^***^		
Comparison of magnitude				
Symbolic	0.054	0.389^***^	0.065	
Non-symbolic	0.025	0.271^**^	0.314^**^	0.318^**^

* *p < 0.05*;

** *p < 0.01*;

*** *p < 0.001*.

### Mean Performance by Gender and Grade

If differentiated by gender, the Mann–Whitney tests indicated that the girls scored significantly higher (See Table 2) in the symbolic test of the Spanish second graders (*U* = 1,055, *p* = 0.02, *r* = −0.26), but this was not the case for the Chilean sample (*p* = 0.727). However, the effect size of the significant differences ranged from low to moderate. Regarding the non-symbolic test, the results were similar, with girls outperforming the boys in the Spanish sample (*U* = 1,103, *p* = 0.42, *r* = −0.23), but not in the Chilean sample (*p* = 0.092). In this case, the effect size was lower than in the symbolic test. The differences detected in the sample disappear in the third grade. In terms of the number of mistakes made on both tests, no significant differences were found between genders in either country.

In the third-grade sample, significant differences were only found between genders for the mathematical fluency test in the Chilean sample (*U* = 1,369, *p* = 0.023, *r* = 0.27). Here, the girls had lower scores (*Md* = 9) than the boys (*Md* = 13). In terms of the number of mistakes made on both tests, no significant differences were found between genders in either country at the third grade level.

### Ratio of Boys to Girls at Various Percentiles

Four groups for each sample were formed according to the scores they obtained and their corresponding percentiles. Group 1 scored in the 1–25th percentile; group 2 in the 26–50th percentile; group 3 in the 51–75th percentile; and group 4 in the 76th percentile or higher. χ^2^ and its significance were calculated to examine significant differences in the ratio of males and females in the different percentile groups. As no significant differences were found in terms of performance of boys and girls in the third-grade tests (except for mathematical fluency, which had a low effect size), the groups were formed by students from both countries. In the case of the second graders, the calculations varied by country.

For the third group, there were no significant gender differences in terms of the ratio of boys to girls, the different percentile groups, or for any of the tests (see Table 3). However, significant differences were found in the symbolic test for the Spanish sample of second graders (χ^2^ = 9.65, *p* = 0.022). The percentages were different for the first group (scorings between the 1–25th percentile; χ^2^ = 6.54, *p* = 0.011), with a larger quantity of boys (30.4%) than girls (9.8%). Significant differences were not found in the Chilean sample of second graders.

**Table 3:** Percentage and comparison of third grade boys and girls by groups corresponding to the 25th, 50th, 75th, and 100th percentile.

	**Group 1(P25)**	**Group 2 (P50)**	**Group 3 (P75)**	**Group 4 (P100)**	**Differences between groups**	
					**χ^2^**	***P***
PreDisCal						
Sentences						
Male	30,0	20,9	29,1	20,0	3.80	0.284
Female	21,8	29,5	23,1	25,6		
*N*	61	44	39	44		
Calculation						
Male	32,7	20,0	23,6	23,6	2.39	0.495
Female	32,1	28,2	16,7	23,1		
*N*	61	44	39	44		
Mathematical fluency						
Male	24,5	29,1	22,7	23,6	1.60	0.659
Female	32,1	29,5	19,2	19,2		
*N*	52	55	40	41		
Magnitude comparison						
Symbolic						
Male	27,3	32,7	17,3	22,7	7.10	0.069
Female	24,4	21,8	33,3	20,5		
*N*	61	44	39	44		
Non-symbolic						
Male	27,3	18,2	30,9	23,6	5.65	0.130
Female	26,9	32,1	25,6	15,4		
*N*	61	44	39	44		

*Groups are organized by percentiles: 1–25th percentile (Group 1), 26–50th percentile (Group 2), 51–75th percentile (Group 3), and 76–100th percentile (Group 4)*.

## Discussion

This study set out to compare the basic mathematical abilities of boys and girls, which prove essential for the acquisition of complex math skills. The sample was composed of boys and girls from two Spanish-speaking yet culturally different countries in the second and third grade, ages at which the gender gap starts to appear. The results allow us to conclude, in keeping with the scientific literature on the topic, that in terms of basic mathematical knowledge, boys and girls show no significant differences in mathematical performance. Previous investigations, including various meta-analyses (Lachance and Mazzocco, 2006; Hyde et al., 2008; Lindberg et al., 2010; Voyer and Voyer, 2014; Scheiber et al., 2015), have noted as much. Similarly, Hutchison et al. (2019) found no differences in numerical and magnitude comparison, numerical ordering, magnitude estimation, multiplication, and division, although the population analyzed for their study hailed from the Netherlands, meaning that results could be different in countries with a higher gender gap. The results of this study, however, reveal no significant differences in most of the tasks considered for two Spanish-speaking samples from Chile and Spain, despite the high gender gap detected in international tests for the two countries (such as TIMSS 2019).

The main differences are found in the symbolic test (comparison of two numbers to determine which one is higher) in the Spanish sample of second graders. In particular, the girls performed better and the boys showed a greater variability. Additionally, females had a higher mean performance, and a greater ratio of males fell below the 25th percentile. However, the effect size is small in second grade, and no such results were found in the third grade sample. For the non-symbolic comparison test, the girls performed better once again, although this difference disappeared in third grade. The direction of these gender differences goes against the hypothesis of higher performance in males. In line with these results, previous studies have shown gender differences in favor of females in basic abilities (for instance, Halpern and Wright, 1996).

The only difference in favor of males was found in the Chilean sample in average performance for mathematical fluency in the third grade. Differences in addition and subtraction have been found previously, although there has not always been evidence of these differences (Hutchison et al., 2019). In this case, the effect size was small and no differences were found for calculation, meaning that caution is critical when interpreting the results. In the case of the Chilean third graders, test abilities were recently acquired, and the differences would likely disappear with practice. Importantly, mathematical fluency is decisive for the robust development of complex calculation and is an ability that can be practiced until it is learned (Royer et al., 1999).

Prior studies have indicated that differences in mathematical performance rely on the kind of task students are asked to complete. These studies have found that females perform better in arithmetic and calculus, while males perform better in mathematical problem solving (Byrnes and Takahira, 1993). However, there have also been conflicting results in the research. For instance, Royer et al. (1999) identified that males perform better in math-fact retrieval than females. In another study, Winkelmann et al. (2008) showed that females had poorer basic skills, while males had a slight advantage in mathematical problems, such as equations containing a missing number. The authors explained that the results depended on basic elementary skills and on how math problems are defined, i.e., on the cognitive resource that each task demanded.

One hypothesis is that gender differences can be found in complex areas of mathematics that demand more cognitive resources. In this sense, for example, the literature suggests a relationship between spatial abilities and gender differences in mathematical performance. However, a recent study did not confirm these results and instead concluded that gender differences may depend on the test and the strategy used to solve each item (Ramírez-Uclés and Ramírez-Uclés, 2020). The results support this hypothesis. In particular, the calculation is the task that involves the highest cognitive demand, but no gender differences were found. Another possibility is that gender differences in mathematical performance emerge later in more complex mathematical tasks or are influenced by cultural or social stereotypes, although the results show that at least lower elementary school children do not exhibit such differences.

Another hypothesis for gender differences in mathematical performance is related to the way in which the answer options are presented on tests. For instance, in a study with PISA 2000 data, Lafontaine and Monseur (2009) found that in all the countries analyzed, open-ended (as opposed to multiple choice) answers for reading tasks favored females. For mathematics, Routitsky and Turner supported these conclusions using PISA 2003 data but pointed out that this difference disappeared as the complexity of items increased (Routitsky and Turner, 2003). Future investigations should assess how the questions and type of answer options influence the gender gap.

Previous studies have revealed that the learning routine shows no differences between genders, although boys and girls do express different attitudes toward mathematics (Barbero-García et al., 2007). This could explain why differences can be found in higher grades despite being absent when basic math skills are evaluated. Importantly, gender differences may well be related to the wording of tasks or instructions, or social behaviors that influence how parents, teachers, and even female students view their own mathematical ability, not by any intrinsic math difficulties (see Lindberg et al., 2010).

This study presents several limitations. All the tasks considered had a time limit, which can influence the results. Processing speed is essential for school performance (Dodonova and Dodonov, 2012), and it is important for the diagnosis of learning disabilities, since children with a diagnosis are identical to those with mean performance if we provide them sufficient time (Jordan and Montani, 1997). The second constraint of this study is that no information was obtained on the socioeconomic level of the children. However, both samples attended public schools, and none were disadvantaged. Lastly, this study is exploratory, and despite having a large sample, it cannot be deemed representative of the total population of both countries.

Gender differences in mathematical performance have been less studied in Spanish-speaking countries. This study was laid out as an initial approach to researching this population. Based on existing literature and the results, gender differences vary by task, strategies used to solve problems, cohort, age, and instructions provided. For future research, the sample should be expanded geographically for higher representativeness in relation to social, educational, and cultural differences. Furthermore, it would be interesting to expand the number of courses evaluated to observe the pattern between genders and to include more complex tasks; this should allow specific mathematical areas to be identified in which gender differences can easily be detected (Chacón-Moscoso et al., 2019). These new developments would potentially reveal differences related to the country, factorial invariance, differential item functioning (DIF), and the influence of open-ended (as opposed to multiple choice) questions (Chacón-Moscoso et al., 2016).

### Practical Value, Implications for Educational Intervention

It should be noted that the tests applied in this study could be used for an early diagnosis of gender difference, and with respect to children with low mathematical performance.

## Conclusions

In conclusion, results emphasizing that males outperform females in math do not hold true in the key mathematical areas analyzed with the exception of mathematical fluency in the Chilean sample, where the effect size was small. Therefore, the acquisition of complex abilities should be the same for males and females. At a scientific level and in the news media, care should be exercised when reporting on gender differences because it can influence own opinion of the girls of their mathematical ability. To the best knowledge of the authors, this study is the first to examine gender differences in basic math skills in Spanish-speaking populations that reveals considerable gaps in international studies on the topic.

## Data Availability Statement

The raw data supporting the conclusions of this article will be made available by the authors, without undue reservation.

## Ethics Statement

The studies involving human participants were reviewed and approved by Chilean Commission for Scientific and Technological Investigation (CONICYT, in its Spanish acronym). The protocol was approved by the Ethics Committee of Universidad de la Frontera (Act 066-2017, on Sheet 036-17). The study was in accordance with the standards of the ethical committee of Universidad Autónoma de Chile and with the agreement between the Region of Murcia's Comunidad Autónoma through the Department of Education and Universities, and Universidad de Murcia. Written informed consent to participate in this study was provided by the participants' legal guardian/next of kin.

## Author Contributions

VP, JF-C, and DM conceptualized and designed the study. MS acquired the data. VP, JF-C, and SC-M analyzed and interpreted the data. VP, JF-C, and DM wrote the manuscript. JF-C prepared the figures and tables. SC-M discussed results and reviewed writing. All the authors read and approved the final version of the manuscript.

## Conflict of Interest

The authors declare that the research was conducted in the absence of any commercial or financial relationships that could be construed as a potential conflict of interest.

## Publisher's Note

All claims expressed in this article are solely those of the authors and do not necessarily represent those of their affiliated organizations, or those of the publisher, the editors and the reviewers. Any product that may be evaluated in this article, or claim that may be made by its manufacturer, is not guaranteed or endorsed by the publisher.
